# TRPA1 Has a Key Role in the Somatic Pro-Nociceptive Actions of Hydrogen Sulfide

**DOI:** 10.1371/journal.pone.0046917

**Published:** 2012-10-11

**Authors:** David A. Andersson, Clive Gentry, Stuart Bevan

**Affiliations:** Wolfson Centre for Age Related Diseases, King's College London, London, United Kingdom; Hokkaido University, Japan

## Abstract

Hydrogen sulfide (H_2_S), which is produced endogenously from L-cysteine, is an irritant with pro-nociceptive actions. We have used measurements of intracellular calcium concentration, electrophysiology and behavioral measurements to show that the somatic pronociceptive actions of H_2_S require TRPA1. A H_2_S donor, NaHS, activated TRPA1 expressed in CHO cells and stimulated DRG neurons isolated from *Trpa1^+/+^* but not *Trpa1^−/−^* mice. TRPA1 activation by NaHS was pH dependent with increased activity at acidic pH. The midpoint of the relationship between NaHS EC_50_ values and external pH was pH 7.21, close to the expected dissociation constant for H_2_S (pK_a_ 7.04). NaHS evoked single channel currents in inside-out and cell-attached membrane patches consistent with an intracellular site of action. In behavioral experiments, intraplantar administration of NaHS and L-cysteine evoked mechanical and cold hypersensitivities in *Trpa1^+/+^* but not in *Trpa1^−/−^* mice. The sensitizing effects of L-cysteine in wild-type mice were inhibited by a cystathionine β-synthase inhibitor, D,L-propargylglycine (PAG), which inhibits H_2_S formation. Mechanical hypersensitivity evoked by intraplantar injections of LPS was prevented by PAG and the TRPA1 antagonist AP-18 and was absent in *Trpa1^−/−^* mice, indicating that H_2_S mediated stimulation of TRPA1 is necessary for the local pronociceptive effects of LPS. The pro-nociceptive effects of intraplantar NaHS were retained in *Trpv1^−/−^* mice ruling out TRPV1 as a molecular target. In behavioral studies, NaHS mediated sensitization was also inhibited by a T-type calcium channel inhibitor, mibefradil. In contrast to the effects of NaHS on somatic sensitivity, intracolonic NaHS administration evoked similar nociceptive effects in *Trpa1^+/+^* and *Trpa1^−/−^* mice, suggesting that the visceral pro-nociceptive effects of H_2_S are independent of TRPA1. In electrophysiological studies, the depolarizing actions of H_2_S on isolated DRG neurons were inhibited by AP-18, but not by mibefradil indicating that the primary excitatory effect of H_2_S on DRG neurons is TRPA1 mediated depolarization.

## Introduction

Hydrogen sulfide (H_2_S) is produced endogenously in neuronal and non-neuronal cells from the amino acid L-cysteine by the enzymes cystathionine-β-synthase and cystathionine-γ-lyase with a third enzyme, 3-mercaptopyruvate sulfur transferase, also capable of generating H_2_S in neurons [1]–[3]. Although anti-nociceptive actions of H_2_S have been reported [4]–[6], there are many reports that H_2_S can potentiate nociception, e.g. in pancreatitis or lipopolysaccharide (LPS) induced inflammation [6], [7]. H_2_S is an irritant when it comes in contact with the eyes and airways, and a pro-nociceptive effect of H_2_S has been demonstrated after intrathecal [8], intraplantar [8], [9] or intracolonic [10] administration.

One potential site of action for H_2_S is peripheral sensory neurons and administration of H_2_S can elevate circulating substance P (SP) levels [11]. The H_2_S-evoked increase in SP concentration has been attributed to release from capsaicin-sensitive sensory neurons as it can be reduced by functional desensitization of sensory nerves by pre-treatment with capsaicin [12]–[14]. The molecular basis for the pro-nociceptive effects of H_2_S is unclear. A role for TRPV1 expressed by nociceptive sensory neurons has been proposed based on the observations that H_2_S evoked release of substance P from sensory nerve preparations was reduced by the TRPV1 antagonists, ruthenium red, capsazepine and SB366791 [13]. Similarly, TRPV1 antagonists inhibited neurally mediated secretion and sensory neuron firing evoked by H_2_S in the intestine [15]. T-type voltage gated calcium channels have also been implicated in the pro-nociceptive effects of H_2_S. Intraplantar, intrathecal or intestinal administrations of H_2_S cause mechanical hypersensitivity that can be reduced by the T-type calcium channel inhibitor, mibefradil [7]–[10], or by treatment with antisense oligonucleotides directed against the T-type calcium channel isoform, Ca_v_3.2 [8]. H_2_S sensitizes T-type calcium channels in neurons [8] and potentiation of T-type Ca^2+^ channel currents is likely to lead to increased sensory neuron excitability by facilitating repetitive firing and thereby producingpro-nociceptive effects [16].

H_2_S activates TRPA1 [17], a TRP channel expressed by a sub-population of TRPV1 and neuropeptide-containing sensory neurons [18]–[21]. In the current study we examined the effects of the H_2_S donor, NaHS, on heterologously expressed TRPA1 and isolated sensory neurons, and determined the role of TRPA1 in the behavioral effects elicited by local administration of H_2_S donors. Our study demonstrates that NaHS depolarizes sensory neurons by activating TRPA1 and that the somatic pro-nociceptive effect of NaHS is absent in mice lacking functional TRPA1 but is not affected by the absence of TRPV1. We further show that mechanical hypersensitivity induced by local administration of LPS is prevented by an inhibitor of H_2_S production and lost completely in *Trpa1^−/−^* mice. In contrast the nociceptive effects of intra-colonic administration of NaHS are similar in wild-type and *Trpa1^−/−^* mice. We propose that H_2_S activation of TRPA1 is the major mechanism for the excitation of somatic nociceptive sensory neurons.

## Materials and Methods

### Cell culture

DRG neurons were prepared from adult male or female mice or male Wistar rats using methods described previously [22]. Isolated neurons were cultured in MEM supplemented with 10% fetal bovine serum, 100 U/ml penicillin, 100 µg/ml streptomycin, 2 mM L-glutamine and 50 ng/ml NGF (Promega, Southampton, UK) for less than 24 hours before experimentation.

Untransfected CHO cells and CHO cells expressing mouse TRPA1 were grown in MEM-α medium supplemented with penicillin (100 U/ml), streptomycin (100 µg/ml), L-glutamine (2 mM) and FCS (10%). All media, serum and antibiotics were from Invitrogen (Paisley, UK).

### Intracellular [Ca^2+^] measurements

#### Sensory neuron studies

DRG neurons were loaded with 2 µM Fura-2 AM (Molecular Probes, Paisley, UK) in the presence of 1 mM probenecid for ∼1 hr. The dye loading and subsequent experiments were performed in a physiological saline solution containing (in mM) 140 NaCl, 5 KCl, 10 glucose, 10 HEPES, 2 CaCl_2_, and 1 MgCl_2_, buffered to pH 7.4 with NaOH. Compounds were applied to cells by local continuous microperfusion of solution through a fine tube placed very close to the cells being studied. TRP channel expression in individual neurons was tested functionally by sequential application of agonists for TRPA1 (allyl isothiocyanate, AITC, 50 µM) and TRPV1 (capsaicin, 1 µM). Experiments were conducted at room temperature except where noted in the text. Images of a group of cells were captured every 2 sec using 340 and 380 nm excitation wavelengths with emission measured at 520 nm with a microscope based imaging system (PTI, New Jersey). Analyses of emission intensity ratios at 340 nm/380 nm excitation (R, in individual cells) were performed using the ImageMaster suite of software.

#### 96 well plate assays

Changes in intracellular calcium ([Ca^2+^]_i_) in response to agonists were determined in TRPA1 expressing CHO cells using a Flexstation 3 (Molecular Devices). Cells grown in 96 well black walled plates (Costar, Tewksbury, MA) were loaded with Fura 2-AM at 37°C for 1–1.5 hours and assays were carried out at 25°C. Basal emission ratios (340 nm/380 nm) were measured and changes in ratio determined at various times after compound addition.

### Electrophysiology

DRG neurons and TRPA1 CHO cells were studied under voltage-clamp conditions using an Axopatch 200B amplifier and pClamp 10.0 software (Molecular Devices, Sunnyvale, CA). Whole cell recordings were performed at a holding potential of −60 mV using an extracellular solution with the composition described above for [Ca^2+^]_i_ measurements. Borosilicate glass pipettes (2–5 MΩ, 75–80% series resistance compensation) were filled with (in mM) 140 KCl, 1 CaCl_2_, 2 MgATP, 10 EGTA, and 10 HEPES buffered to pH 7.4 (KOH). This K+-based solution was also used to superfuse the intracellular face of inside-out patches. Inside out and cell attached patches were recorded using a Ca^2+^-free solution containing (in mM) 140 NaCl, 5 KCl, 1 MgCl_2_, 10 HEPES and 1 EGTA, pH 7.4 (NaOH) in the pipettes and the same solution was used as bath solution for the cell attached configuration. DRG neurons were studied using an intracellular solution containing (in mM) 140 CsCl, 1 CaCl_2_, 2 MgATP, 10 EGTA and 10 HEPES, pH 7.4 (CsOH). Current-clamp recordings of DRG neurons were performed in an extracellular solution containing (in mM) 140 NaCl, 3 KCl, 2CaCl_2_, 1MgCl_2_, 10 HEPES and 10 glucose, pH 7.4 (NaOH). Drugs were applied by local microperfusion with a rapid solution changer (RSC-200, Biologic, Claix, France).

### Behavioral experiments

All animal studies were carried out according to U.K. Home Office Animal Procedures (1986) Act. Data shown are from male and female C57Bl/6J mice, homozygote *Trpa1* ^−/−^ and *Trpa1* ^+/+^, and *Trpv1* ^−/−^ and *Trpv1* ^+/+^ littermates. The Trpa1-null mice and wild-type littermates were bred from heterozygotic mice provided by Drs. Kelvin Kwan (Harvard Medical School, Boston, MA) and David Corey (Harvard Medical School, Boston, MA) (Kwan et al, 2006). The Trpv1-null mice and wild-type littermates were kindly provided by Professor Sue Brain (King's College London, UK).

Mechanical thresholds were measured using an Analgesymeter (Ugo-Basile, Milan). Mice were kept in their holding cages to acclimatize (10–15 min) to the experimental room. The experimenter then lightly restrained the mouse and applied a constant increasing pressure stimulus to the dorsal surface of the hind paw using a blunt conical probe. The nociceptive threshold was defined as the force in grams at which the mouse withdrew its paw. In order to avoid tissue injury a 150 g force cut-off value was used.

Cold sensitivity was assessed by measuring the time for paw withdrawal from a 10°C cold plate (Ugo Basile, Milan) of lightly restrained mice [23].

NaHS (0.1 and 1 nmole in 25 µl saline), L-cysteine (100 nmole in 10 µl saline) and LPS (0.1–10 µg in 25 µl saline) were injected subcutaneously into the plantar surface of one of the hind paws using a 50 µl luer-syringe (Hamilton Co.) fitted with a 26-gauge×3/8 inch intradermal needle. AP-18 (25 nmole, Maybridge, Tintagel, UK) was made up in 1% DMSO/0.5% Tween 80/saline and co-administered with vehicle or NaHS by intraplantar injection in a volume of 25 µl. Propargylglycine (PAG, 11..25 mg/kg) was dissolved in saline and injected intraperitoneally in a volume of 0.2 ml.

### Behavioural responses to intra-colonic NaHS

Using a cannula with a rounded tip, 5 nmoles NaHS in 50 µl saline or vehicle alone was instilled into the colon at 3 cm from the anus in the mice, with application of Vaseline in the perianal area. The chosen dose of NaHS has previously been shown to evoke a maximal pronociceptive effect in mice [10]. Immediately after the intra-colonic instillation, the number of visceral pain-related nociceptive behaviors was observed and counted for 15 min. The behaviors defined as pain-related were (a) licking of the abdomen, (b) stretching the abdomen, (c) squashing of the lower abdomen against the floor and (d) abdominal retractions [24]. Referred hypersensitivity was assessed by determining the frequency of responses to stimulation of the abdomen with von Frey hairs prior to (baseline) and 15–30 min after intra-colonic administration of NaHS. The lower to mid abdomen of the mice was stimulated mechanically by three von Frey filaments with strengths of 0.02, 0.16 and 1.0 g, in the ascending order of strength, at intervals of 5–10 s, 10 times for each filament. Stimulation was concentrated on the lower to mid abdomen avoiding the area of the external genitalia. The following behaviors were taken as a withdrawal response: (a) sharp retraction of the abdomen; (b) immediate licking or scratching at the site of stimulation; (c) jumping.

### Drugs and chemicals

Mibefradil was from Tocris (Bristol, UK). Unless stated otherwise, salts and other reagents were from Sigma-Aldrich (Poole, UK).

## Results

### Effects of H_2_S on sensory TRP channels

**Figure 1:**
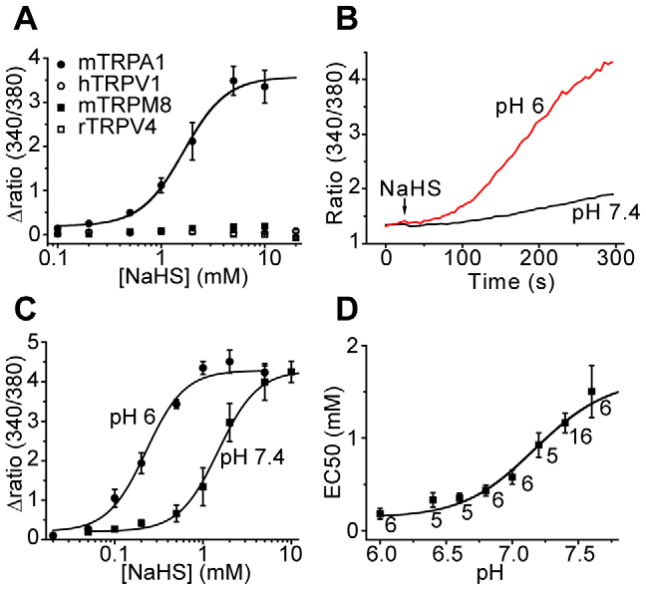
NaHS activation of TRPA1. A) NaHS evoked a concentration-dependent increase in [Ca^2+^]_i_ in Fura-2 loaded mTRPA1 expressing CHO cells but not in cells expressing TRPV1, TRPM8 or TRPV4 (mean ± s.e.m. , n = 3 for each point). B) Potentiation of NaHS evoked Ca^2+^ response at lower external pH. C) NaHS concentration-Ca^2+^ response relationships at physiological and acidic external pH showed a reduction in EC_50_ value at pH 6 (each point is mean ± s.e.m of triplicate samples). D) Relationship between external pH and EC_50_ values for NaHS activation of TRPA1. Logistic curve fitted to the data has a mid-point at pH 7.21. The slope factor for the relationship between [H+] and EC_50_ values was 1.86±0.46. Each point is mean ± s.e.m of EC_50_ values from between 5–16 individual concentration-response curves for each pH value.

The selectivity of H_2_S action on sensory TRP channels was first investigated by examining the effects of the H_2_S donor, NaHS, on heterologously expressed TRP channels using increases in intracellular calcium concentration ([Ca^2+^]_i_) as an index of channel activation. NaHS evoked a concentration dependent increase in [Ca^2+^]_i_ in mTRPA1 expressing CHO cells with a mean EC_50_ value of 1.06±0.08 mM (n = 15) at pH 7.4 (Fig. 1A), which is in close agreement with the results from our previous study [17]. In contrast, no significant [Ca^2+^]_i_ response was evoked in cells expressing TRPV1, TRPV4 or TRPM8 at concentrations of NaHS up to 20 mM (Fig. 1A).

NaHS is in equilibrium with H_2_S and at physiological pH about 30% of the added NaHS exists as H_2_S. Although H_2_S readily permeates membranes by diffusion through the lipid phase [25], it dissociates in aqueous solution to form the poorly membrane permeant ions H^+^ and HS^−^ with a pK_a_ of 7.04. We therefore determined the effect of extracellular pH on the agonist effects of added NaHS. Alkaline solutions alone activated TRPA1 [26] which precluded investigations at >pH 7.6. But as shown in Fig. 1B the response to 500 µM NaHS was greatly enhanced at pH 6 when compared to the response at pH 7.4. This effect was reflected in a reduction of the EC_50_ value at the lower extracellular pH (Fig. 1C). Concentration response curves at different pH values demonstrated a clear relationship between the EC_50_ value and pH with a mid-point at pH 7.21 (Fig. 1D). Activation of TRPA1 was evoked by external concentrations of NaHS below 100 µM in experiments with acidic external solutions.

**Figure 2:**
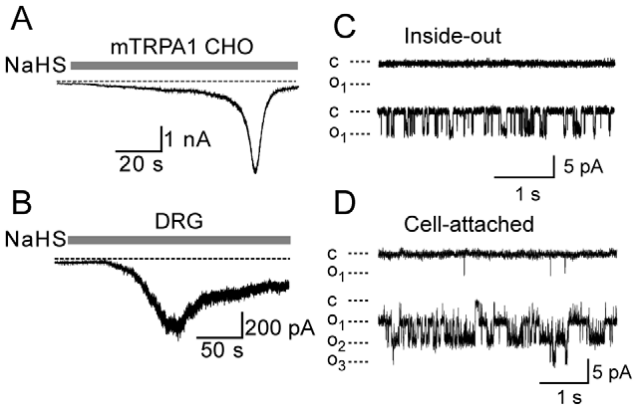
Membrane currents evoked by NaHS in TRPA1 expressing cells. Whole cell currents evoked by 5 mM NaHS in A) TRPA1 CHO cell and B) DRG neuron. TRPA1 expression in DRG neurons was confirmed by subsequent activation by AITC. C) Single channel currents evoked by a relatively low concentration of NaHS (100 µM) applied to the intracellular side of an inside-out membrane patch from a TRPA1 CHO cell. D) NaHS evoked single channel currents in a cell-attached patch evoked by extracellularly applied NaHS (2 mM).

In electrophysiological studies NaHS activated TRPA1 heterologously expressed in CHO cells as well as natively expressed TRPA1 in DRG neurons. NaHS evoked a characteristic TRPA1 mediated whole cell current with a slow increase in inward current followed by an accelerating phase of current growth followed by pronounced inactivation (Fig. 2A, B). No currents were evoked in untransfected CHO cells. We also examined the ability of NaHS to activate TRPA1 in membrane patches. Addition of NaHS activated single channel currents in inside-out membrane patches from TRPA1 CHO cells (Fig. 2C). TRPA1 activation by NaHS was also evident in single channel recordings using cell attached patches with bath application of NaHS, which indicates that the agonist effects require membrane permeation, since the recorded channels are not in direct contact with the bath solution in this configuration (Fig. 2D). Relatively low concentrations of NaHS were sufficient to stimulate TRPA1 in the inside-out configuration (e.g. 100 µM in Fig. 2C) further suggesting that H_2_S stimulates TRPA1 at an intracellular site.

### NaHS stimulates TRPA1 expressing DRG neurons

**Figure 3:**
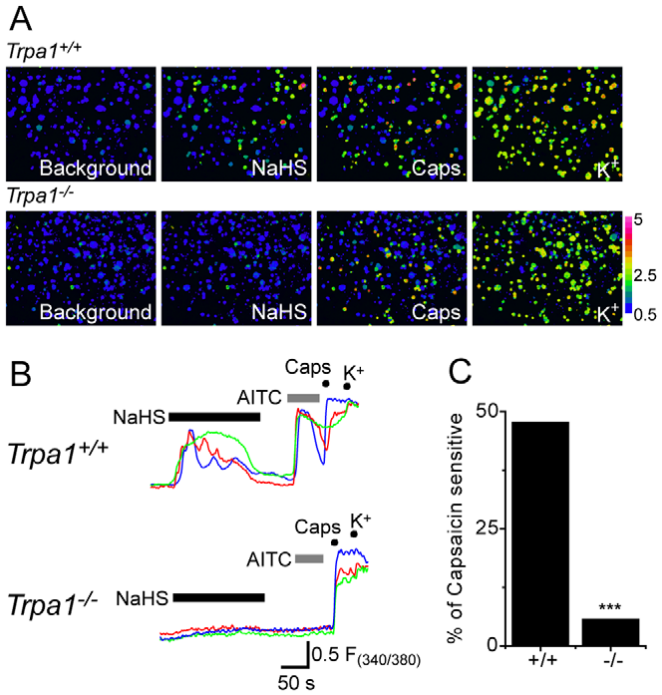
NaHS activation of DRG neurons is mediated by TRPA1. A) Pseudocoloured images of isolated mouse DRG neurons loaded with Fura-2 sequentially challenged with NaHS and capsaicin and depolarized by a high K^+^ solution NaHS increased [Ca^2+^]_i_ in a subset of capsaicin-sensitive neurons from wild-type mice (top) but not in neurons from *Trpa1* ^−/−^ mice Bottom). B) Fura-2 responses showing NaHS-evoked increases in [Ca^2+^]_i_ in DRG neurons from *Trpa1* ^−/−^ and wild-type mice. NaHS evoked clear responses in AITC-sensitive neurons from wild-type mice, but either no response or very slow, small responses in *Trpa1* ^−/−^ DRG neurons. C) Percentage of capsaicin-sensitive DRG neurons from *Trpa1* ^−/−^ and wild-type mice that responded to NaHS.

To investigate the agonist effects and selectivity of NaHS on native cells, we examined increases in [Ca^2+^]_i_ in DRG neurons exposed first to NaHS and then sequentially to the TRPA1 and TRPV1 agonists allyl isothiocyanate (AITC) and capsaicin. NaHS evoked an increase in [Ca^2+^]_i_ in 42% (87/207) of capsaicin-sensitive DRG neurons isolated from wild-type mice. There was a close correspondence between neurons that responded to NaHS and to AITC (Fig. 3B, top), although not all AITC-sensitive neurons showed a clear response to NaHS. In addition, a small population (6%, 5/78) of capsaicin-sensitive DRG neurons that were AITC-insensitive showed small responses to NaHS, but these were unlike the responses seen in the majority of NaHS-sensitive neurons. These findings are consistent with those recently reported in two independent studies of NaHS effects on sensory neurons [27], [28].

We examined the importance of TRPA1 for the NaHS evoked [Ca^2+^]_i_ responses in Fura-2 loaded DRG neurons isolated from *Trpa1* ^−/−^ mice and wild-type littermates. NaHS sensitivity was greatly reduced in the *Trpa1^−/−^* neurons (Fig. 3B, C). In these experiments, 47.7% (244/511) of capsaicin-sensitive DRG neurons from *Trpa1^+/+^* mice responded to NaHS, whereas only 5.7% (30/527) of the capsaicin-sensitive DRG neurons from TRPA1-deficient mice responded to NaHS. In addition, the observed increases in [Ca^2+^]_i_ in *Trpa1^−/−^* DRG neurons were much smaller than those seen in neurons from wild-type, *Trpa1^+/+^*, littermate mice (Fig. 3B). These residual responses in *Trpa1^−/−^* neurons were similar to those noted in AITC-insensitive DRG neurons from wild-type mice. The data were therefore consistent with the hypothesis that the major excitatory effect of H_2_S on DRG neurons is mediated by TRPA1.

### Role of TRPA1 in the somatic pro-nociceptive effects of H_2_S *in vivo*

**Figure 4:**
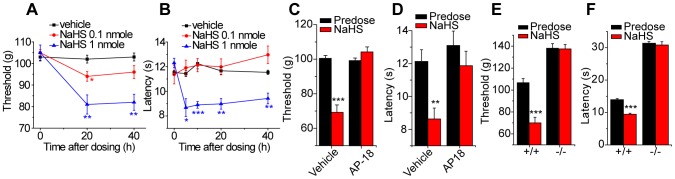
The pronociceptive effects of NaHS require TRPA1. Intraplantar administration of NaHS (1 nmole) reduced A) mechanical paw pressure threshold and B) latency for paw withdrawal from a cold (10°C) plate stimulus in wild type C57Bl/6 mice (n = 5 per group). NaHS (1 nmole intraplantar) evoked mechanical hypersensitivity (C) and cold hypersensitivity (D) were inhibited by intraplantar co-administration of TRPA1 antagonist AP-18 (25 nmole, n = 6) in wild-type mice and were absent in *Trpa1* ^−/−^ mice, n = 6 (E, F).

A pro-nociceptive effect of intraplantar administration of H_2_S has been demonstrated using paw pressure thresholds as an end-point [8], [9]. These earlier studies pointed to an important role of T-type calcium channels in the pro-nociceptive effects of H_2_S. As our in vitro studies indicated that TRPA1 was important for sensory neuron activation, we used this method to assess the role of TRPA1 for the behavioral effects. Intraplantar administration of 0.1–10 nmole NaHS evoked a dose-dependent marked mechanical hypersensitivity in C57Bl/6 mice (Fig. 4A). The effect of NaHS was not restricted to mechanical responses and an increase in cold sensitivity was noted after intraplantar administration of 1 nmole NaHS in C57Bl/6 mice (Fig. 4B). NaHS-evoked mechanical and cold hypersensitivities were inhibited by intraplantar co-administration of the TRPA1 antagonist, AP-18 (Fig. 4C, D). In addition, intraplantar administration of NaHS evoked mechanical (Fig. 4E) and cold (Fig. 4F) hypersensitivities in wild-type, *Trpa1^+/+^* mice that were absent in *Trpa1^−/−^* mice (Fig. 4E, F).

**Figure 5:**
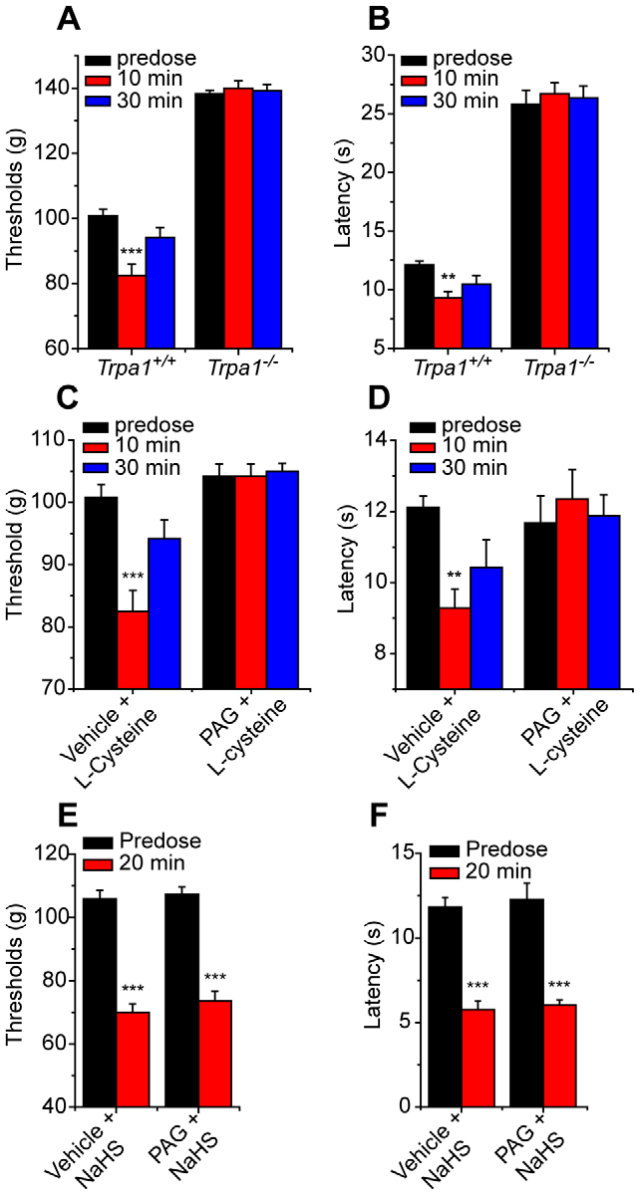
TRPA1 is required for the pro-nociceptive effects of cysteine. Intraplantar administration of L-cysteine (100 nmole) reduced A) mechanical paw pressure threshold and B) latency for paw withdrawal from a cold plate stimulus in wild type mice (n = 6 per group). Cysteine-evoked mechanical hypersensitivity (C) and cold hypersensitivity (D) were inhibited by systemic administration of a cystathionine β-synthase inhibitor (PAG, 11.25 mg/kg i.p. 60 minute pretreatment.), but PAG was without effect on NaHS-evoked mechanical (E) and cold (F) hypersensitivities (n = 6 per group).

H_2_S can be generated enzymatically from L-cysteine. We therefore investigated the effects of local intraplantar administration of L-cysteine on mechanical and cold sensitivities. The results were identical to the effects of intraplantar NaHS injection (Fig. 5A, B). L-cysteine evoked both cold and mechanical hypersensitivities in *Trpa1^+/+^* mice but not in evoked responses in wild-type mice were inhibited by prior, local injection of the cystathionine β-synthase inhibitor D,L-propargylglycine (PAG, 11 mg/kg, Fig. 5C, D). To determine whether the effect of PAG could be explained by an inhibitory effect on sensory neuron function, we examined if PAG could inhibit the response evoked by H_2_S. PAG did not inhibit the sensitizing actions of NaHS as increases in both mechanical and cold sensitivities were evoked by NaHS in PAG- and vehicle-treated wild-type mice (Fig. 5E, F).

**Figure 6:**
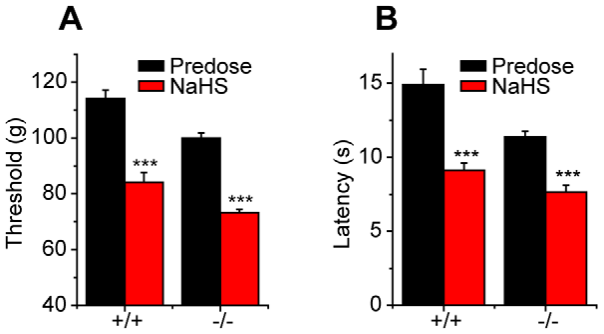
NaHS-induced hypersensitivity is independent of TRPV1. Intraplantar administration of NaHS (1 nmole) evoked similar mechanical (A) and cold (B) hypersensitivities in *Trpv1* ^−/−^ and wild-type mice (n = 6 per group).

As TRPV1 has been proposed to play a major role in the actions of H_2_S, we investigated the pro-nociceptive effects of NaHS in *Trpv1^−/−^* and wild-type mice using both mechanical and cold sensitivities as readouts. In contrast to the differences seen in *Trpa1^−/−^* mice, we found that both mechanical (Fig. 6A) and cold (Fig. 6B) hypersensitivities developed similarly in *Trpv1^−/−^* and *Trpv1^+/+^* mice after intraplantar administration of NaHS.

### TRPA1 is not required for NaHS-evoked visceral hypersensitivity

**Figure 7:**
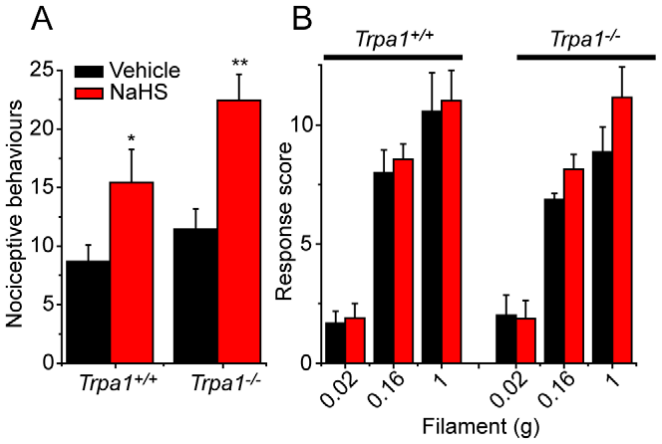
The pronociceptive effect of visceral NaHS is independent of TRPA1. A) The number of pain-related behaviors produced by intracolonic administration of NaHS (5 nmoles) or vehicle did not differ between *Trpa1* ^−/−^ and *Trpa1* ^−/−^ mice (n = 7–9). B) Compared to vehicle, intracolonic NaHS did not evoke referred hyperalgesia, measured as the number of withdrawal responses produced by abdominal stimulation with von Frey filaments, in *Trpa1* ^−/−^ and *Trpa1* ^−/−^ mice (n = 7–9).

Intracolonic instillation of NaHS has previously been shown to evoke a visceral nociceptive response associated with abdominal mechanical hyperalgesia [10]. To assess the contribution of TRPA1 to NaHS induced visceral nociception, we examined the behavioral effects of intracolonic administration of NaHS (5 nmole) and vehicle in *Trpa1^+/+^* and *Trpa1^−/−^* mice (Fig. 7A, B). In contrast to the results obtained with intraplantar injections of NaHS, intracolonic administration of NaHS produced identical nociceptive effects in *Trpa1^+/+^* and *Trpa1^−/−^* mice, suggesting that although TRPA1 stimulation is essential for the somatic pronociceptive effects of H_2_S, it is not required for the visceral effects of H_2_S. It is worth noting that administration of vehicle itself produced a significant nociceptive response, as reported by others [10], [29]. Previous studies have demonstrated a referred mechanical hyperalgesia following colonic instillation of NaHS in mice [10], [29]. Here, we found no difference between the sensitivity to abdominal stimulation with von Frey filaments between mice treated with vehicle and NaHS and the mechanical sensitivity was virtually identical in *Trpa1^+/+^* and *Trpa1^−/−^* mice (Fig. 7B).

### Do T-type calcium channels play a role in NaHS evoked hypersensitivity?

**Figure 8:**
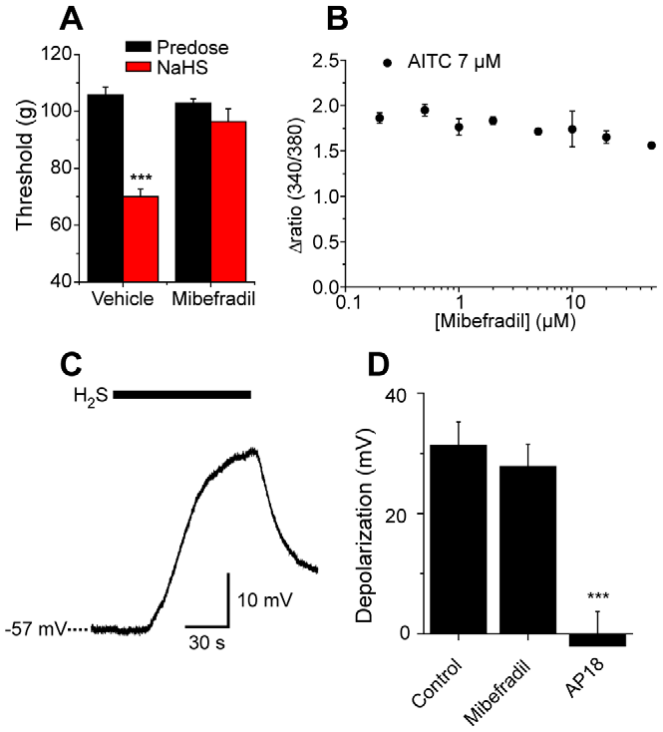
T-type calcium channels play a role in NaHS-evoked hypersensitivity but TRPA1 mediates DRG neuron depolarization. A) T-type calcium channel blocker, mibefradil (9 mg/kg i.p.) inhibited intraplantar NaHS-evoked mechanical hypersensitivity (n = 7 per group). B) Mibefradil (10 µM) did not antagonize TRPA1 activation evoked by AITC in TRPA1 CHO cells. C) NaHS (5 mM) depolarized a subset of DRG neurons. D) NaHS depolarization of DRG neurons was blocked by the TRPA1 antagonist AP-18 (10 µM) but not by mibefradil (10 µM).

Other investigators have proposed that the pro-nociceptive effects of H_2_S are mediated by potentiation of T-type calcium channel activity [7]–[10]. We therefore re-examined the effects of T-type channel inhibition on NaHS evoked mechanical hypersensitivity. In agreement with previous reports we found that the T-type channel inhibitor mibefradil inhibited the pro-nociceptive effects of NaHS. The reduction in paw pressure thresholds evoked by NaHS in wild-type mice was greatly reduced by prior administration of mibefradil (Fig. 8A).

One possible explanation for the *in vivo* actions of mibefradil was that it acted as a TRPA1 antagonist in addition to its actions on T-type calcium and voltage gated sodium channels [30]. The effect of mibefradil was therefore examined using TRPA1 expressing CHO cells. Mibefradil (up to 50 µM) did not act as an TRPA1 agonist and had no antagonistic effect as it failed to inhibit the increases in [Ca^2+^]_i_ evoked by a sub-maximally active (∼EC_80_) concentration of AITC (Fig. 8B). Next we examined the effects of mibefradil on the responses of DRG neurons. Voltage recordings rather than voltage clamp measurements of membrane current were studied so that any voltage dependent effects would not be overlooked. In this study small diameter neurons were selected to enrich for neurons with a nociceptor phenotype. NaHS depolarized 7/10 of the capsaicin-sensitive DRG neurons as illustrated in Fig. 8C. Experiments were also carried out in the presence of the T-type calcium channel inhibitor mibefradil. 10 µM mibefradil had no significant effect on either the number (6/9) of neurons responding to NaHS or the amplitude of the depolarization (Fig. 8D). In contrast, the TRPA1 antagonist, AP-18 (10 µM), significantly inhibited the depolarizing response to NaHS (Fig. 8D).

### Role of H_2_S and TRPA1 in LPS evoked hyperalgesia

**Figure 9:**
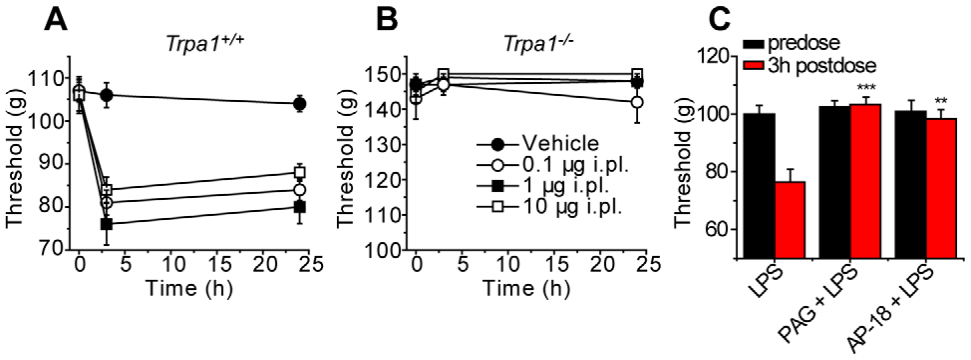
The nociceptive effect of local LPS requires stimulation of TRPA1. Intraplantar administration of LPS (0.1–10 µg) evoked mechanical hyperalgesia in *Trpa1^+/+^* (A) but not in *Trpa1^−/−^* mice (B). Intraplantar injection of 10 µg produced a mechanical hypersensitivity in C57Bl/6 mice (C). Pretreatment with PAG (11.25 mg/kg i.p., 1 h before LPS) or AP-18 (3 mg/kg i.p., 30 min before LPS), completely prevented the development of the LPS-induced mechanical hyperalgesia (** p<0.01, *** p<0.001, ANOVA followed by Tukey's HSD test).

Administration of lipopolysaccharide (LPS) induces mechanical hypersensitivity in mice and H_2_S has been proposed to play an important role in the inflammatory and hyperalgesic effects produced by LPS [6], [31], [32]. To determine the importance of TRPA1 for LPS evoked mechanical hyperalgesia, we injected *Trpa1^+/+^* and *Trpa1^−/−^* mice (i.pl.) with 0.1–10 µg of LPS (Fig. 9A, B). LPS evoked a marked and long-lasting hyperalgesia at all doses tested in *Trpa1^+/+^* mice, but was without any effect on the paw withdrawal thresholds in *Trpa1^−/−^* mice. Administration of the selective TRPA1 antagonist AP18 (3 mg/kg) 30 min before intraplantar LPS injections completely prevented development of the LPS induced hyperalgesia in C57Bl/6 mice (Fig. 9C). Finally, we used the cystathionine β-synthase inhibitor PAG to assess the role of H_2_S production for the LPS-induced, TRPA1 dependent hyperalgesia (Fig. 9C). Similar to our observations with cysteine above (see Fig. 5C, D), PAG (11 mg/kg, 1 h before LPS) completely prevented the development of LPS-induced mechanical hyperalgesia.

## Discussion

Our results extend the original observation that NaHS can stimulate TRPA1 and demonstrate that TRPA1 is required for the pronociceptive effects of H_2_S *in vivo* [17]. Our results also confirm and extend the recent independent findings of Miyamoto and colleagues (2011) that NaHS can activate TRPA1 in DRG neurons.

The pH-dependent agonism of the H_2_S donor NaHS, with lower EC_50_ values at more acidic extracellular pH, is consistent with membrane permeation by H_2_S. This is supported by the finding that the mid-point of the EC_50_-external pH relationship (pH 7.21) was close to the cited pK_a_ (pH 7.04) for the dissociation of H_2_S into the membrane impermeant ions H^+^ and SH^−^. Such pH sensitivity suggests that increased permeation of H_2_S will potentiate neuronal activation as well as non-neuronal intercellular H_2_S signalling in acidic conditions that occur in conditions such as inflammation and ischemia. It is unlikely that the effect of reduced pH is explained by a direct action on TRPA1, since [Ca^2+^]_i_ responses evoked by phenylarsine oxide were similar at pH7.4 and pH6 (data not shown). H_2_S has been proposed as a pro-nociceptive mediator in LPS-induced inflammation [31] and pancreatitis [7], with an increased production of H_2_S reported during inflammatory conditions [31]. Activation of sensory neurons and the consequent release of CGRP are important components of both inflammation [33] and protection from ischemic-reperfusion injury [34], [35]. Our findings that LPS-induced mechanical hyperalgesia relies on activation of TRPA1 and is prevented by inhibition of H_2_S production, suggest that H_2_S-mediated activation of TRPA1 may explain or contribute to the H_2_S dependent effects observed in previous studies of inflammation [7], [31].

An intracellular site of action for H_2_S is consistent with the finding that extracellular application of NaHS activated single channel currents in cell-attached membrane patches. In this configuration, the tight seal between plasma membrane and the glass recording pipette prevents access to the extracellular surface within the membrane patch. H_2_S must therefore cross the membrane to exert its action. Furthermore the finding that intracellular application of a relatively low concentration (100 µM) of NaHS at pH7.4 to the intracellular side of an isolated membrane patch evoked robust TRPA1 single channel activity is consistent with an intracellular site of action for H_2_S. H_2_S is known to modify proteins by S-sulfhydration of proteins by converting cysteine –SH groups to –SSH [36] and intracellular N-terminal cysteine modification is known to activate TRPA1 [37], [38]. The data are therefore consistent with TRPA1 activation following cysteine modification by H_2_S.

NaHS also evoked responses in a population (∼6% of total neurons) of non-TRPA1 expressing DRG neurons from wild-type mice and DRG neurons from *Trpa1* ^−/−^ mice. These responses were smaller than those evoked in TRPA1-expressing neurons. The mechanism underlying these responses is unclear. It is unlikely that TRPV1 activation mediates the increase in [Ca^2+^]_i_ as the great majority of TRPV1-positive, TRPA1-negative DRG neurons failed to respond to NaHS. NaHS also had no agonist effect on TRPV1 expressing CHO cells. H_2_S is known to modulate the activity of other ion channels, for example promoting the opening of K_ATP_ [39] and NMDA channels [40] and inhibiting L-type voltage gated calcium channels [41]. H_2_S may therefore depolarize some DRG neurons by another ion channel mechanism. Although our data show that activation of T-type calcium channels is not the principal mode of H_2_S agonism in DRG neurons, we do not rule out the possibility that this mechanism operates in a small percentage of neurons to increase [Ca^2+^]_i_. It is possible that such a mechanism is of particular importance for the visceral pain and hypersensitivity produced by intracolonic NaHS.

Local, intraplantar administration of either NaHS or L-cysteine evoked both mechanical and cold hypersensitivities. The inhibitory effect of the enzyme inhibitor PAG on the actions of L-cysteine but not NaHS indicates the importance of enzymatic production of H_2_S for the effects of L-cysteine. The development of mechanical hyperalgesia induced by intraplantar injections of LPS was very effectively prevented by PAG and AP-18 and intraplantar LPS was without effect in *Trpa1^−/−^* mice. These findings strongly suggest that H_2_S production and stimulation of TRPA1 are necessary for the pronociceptive actions of local LPS.

During the revision of this manuscript, an independent study reported that the TRPA1 antagonist AP18 reduced the visceral nociceptive and pronociceptive effects evoked by intracolonic NaHS [29]. These results differ from our findings using TRPA1 knockout mice where we failed to establish a role for TRPA1. In our studies, intracolonic NaHS evoked nociceptive behaviours that were greater in magnitude than those observed in vehicle treated mice, However, this nociceptive effect of NaHS was not diminished in mice lacking TRPA1, which indicates that the visceral nociceptive actions of H_2_S are independent of TRPA1. Our results suggest that intracolonic NaHS primarily exerts nociceptive effects through other targets such as T-type calcium channels [42] and not through a TRPA1 mediated mechanism. It is known that mediators released from intestinal cells can stimulate sensory neurons and we cannot rule out the possibility that the sensory effects of instilled H_2_S may involve non-neuronal as well as neuronal mechanisms.

In our studies wild-type and TRPA1-deficient mice responded similarly to abdominal stimulation with von Frey filaments following administration of NaHS. Importantly the hypersensitivity seen after instillation of vehicle was not significantly different to that seen after administration of NaHS. Thus, unlike some other reports [10], [29], NaHS failed to evoke referred mechanical allodynia in our experiments even though nociceptive behaviours were evoked by NaHS. This contrasts with our finding that NaHS evoked TRPA1-dependent mechanical and cold sensitivities when injected into the paw. The reasons for these discrepancies are not clear, but the marked effect of vehicle instillation alone in our visceral studies and differences in the strains of mice used (C57Bl/6J in our study and ddY mice in the other cited studies of intracolonic NaHS) may underlie our failure to detect H_2_S mediated visceral mechanical hypersensitivity.

TRPA1 has been implicated in the behavioral responses to mechanical [43]–[45] and cold [23], [46], [47] stimuli, although the role of TRPA1 as a primary transducer of cold stimuli is contentious [48]–[50]. A recent publication showed that cold augmented the effects of sub-maximal TRPA1 activation by other stimuli [51] which is consistent with cold potentiation of H_2_S activated TRPA1. Conceptually, the behavioral effects of intraplantar H_2_S may be mediated peripherally due to actions on sensory neurons or surrounding tissues or involve central sensitization as a result of increased sensory neuron input to the spinal cord [52], [53]. Irrespective of the mechanisms, our results show that both H_2_S-evoked cold and mechanical hypersensitivities were abrogated by local, peripheral administration of a TRPA1 antagonist or by genetic deletion of TRPA1. These findings can be simply explained if the primary action of H_2_S is to stimulate a sub-population of DRG neurons by TRPA1 activation. Such a conclusion is not at variance with the original conclusion that H_2_S exerted its effects via capsaicin-sensitive sensory neurons, as many TRPV1-expressing neurons also express functional TRPA1 channels.

The pro-nociceptive effects of H_2_S have been attributed to an action on TRPV1 based on the abilities of the TRPV1 antagonists to reduce SP release and sensory neuron firing in airways and intestinal preparations [13], [15]. However, we found that H_2_S evoked hypersensitivities developed normally in *Trpv1^−/−^* mice, which is consistent with our *in vitro* findings that H_2_S does not activate TRPV1 in either DRG neurons or in a heterologous expression system. Our data are in agreement with results from Kawabata and colleagues who found that H_2_S-evoked mechanical hyperalgesia in the paw was not inhibited by the TRPV1 antagonist capsazepine [9]. The reason for the discrepancy between our results and some other previous reports that support a role of TRPV1 is not clear. One possibility is that the importance of TRPV1 in overall neuronal function differs between visceral and somatic afferents. Another possible confounding factor is the specificity of some of the agents when used at high concentrations in previous H_2_S studies [13], [15]. Ruthenium red and BCTC are not selective TRPV1 antagonists and inhibit other TRP channels at the concentrations tested as H_2_S response inhibitors [54], [55]. At high concentrations (10 µM), capsazepine has non-TRPV1 actions such as inhibition of voltage gated calcium channels [56], which would reduce neuropeptide release. AMG9810 can also act at voltage gated calcium and sodium channels at micromolar concentrations [57]. Finally the selectivity of SB366791 as a TRPV1 antagonist is unclear and some data are consistent with inhibitory effects of this compound on channels other than TRPV1 [58].

T-type as well as N-type calcium channels have roles in the transmission of nociceptive signals [16], [59]. Our *in vivo* data agree well with other reports that T-type calcium channel activity is important for the pro-nociceptive effects of intraplantar H_2_S [7]–[10]. The T-type calcium channel inhibitor, mibefradil, inhibited the pro-nociceptive effects of H_2_S *in vivo* but in our experiments had no major role in H_2_S evoked depolarization of isolated DRG neurons. T-type (Ca_v_3.2) calcium channels expressed on the endings of afferent fibers regulate neuronal excitability and promote repetitive action potential firing [16]. H_2_S mediated potentiation of T-type channels is likely to result in enhanced neuronal firing in stimulated neurons and we suggest that TRPA1 and T-type calcium channels act in concert to depolarize (TRPA1) and evoke trains of action potentials (T-type channels) in nociceptive sensory neurons.
